# Leveraging meaning-induced neural dynamics to detect covert cognition via EEG during natural language listening—a case series

**DOI:** 10.3389/fpsyg.2025.1616963

**Published:** 2025-07-08

**Authors:** Ludvik Alkhoury, James O'Sullivan, Giacomo Scanavini, Jin Dou, Joshitha Arora, Lilah Hamill, Abigail Patchell, Ana Radanovic, William D. Watson, Edmund C. Lalor, Nicholas D. Schiff, N. Jeremy Hill, Sudhin A. Shah

**Affiliations:** ^1^Department of Radiology, Weill Cornell Medicine, New York, NY, United States; ^2^Department of Biomedical Engineering, University of Rochester, Rochester, NY, United States; ^3^Child Psychology, Blythedale Children's Hospital, Valhalla, NY, United States; ^4^Department of Biomedical Engineering, Del Monte Institute for Neuroscience, University of Rochester, Rochester, NY, United States; ^5^Department of Neuroscience, Del Monte Institute for Neuroscience, University of Rochester, Rochester, NY, United States; ^6^Department of Neurology, Brain and Mind Research Institute, Weill Cornell Medicine, New York, NY, United States; ^7^National Center for Adaptive Neurotechnologies, Stratton VA Medical Center, Albany, NY, United States; ^8^Department of Electrical and Computer Engineering, State University of New York, Albany, NY, United States

**Keywords:** natural language, temporal response function (TRF), pediatric, disorder of consciousness, cognitive function

## Abstract

At least a quarter of adult patients with severe brain injury in a disorder of consciousness may have cognitive abilities that are hidden due to motor impairment. In this case series, we developed a tool that extracted acoustic and semantic processing biomarkers from electroencephalography recorded while participants listened to a story. We tested our method on two male adolescent survivors of severe brain injury and showed evidence of acoustic and semantic processing. Our method identifies cognitive processing while obviating demands on attention, memory, and executive function. This lays a foundation for graded assessments of cognition recovery across the spectrum of covert cognition.

## 1 Introduction

In at least 25% of adults with severe brain injury who are diagnosed with a disorder of consciousness (DoC) such as vegetative state, functional magnetic resonance imaging (fMRI) and electroencephalography (EEG) can reveal high-level cognitive activity that is otherwise hidden due to profound motor impairment (Bodien et al., 2024).

Covert cognition is currently detected via fMRI and EEG tests that measure brain responses correlated with imagined or attempted movements to command. Brain activity following the command “move your hand” is contrasted against the command “relax”; a positive response indicates the patient's ability to hear, understand, and repeatedly perform the task without loss of attention, drowsiness, or other factors limiting sustained performance (Bodien et al., 2024). This unequivocally signals cognitive-motor dissociation (CMD).

However, the patient must cross a very high threshold of cognitive functioning to achieve a positive response in established CMD testing (Talukdar et al., 2019). Unsurprisingly, these tasks may fail to identify patients in whom cognitive abilities have only partially recovered.

Brain responses to language provide an attractive alternative window into covert cognition. Comprehension of a spoken text—in other words, integration of successive word meanings to form a context—requires attention, lexical access, syntactic parsing, working memory operations and inference, among other tightly integrated cognitive processes (Moore, 2007). As such, it entails many of the elements of cognition—just as command-following paradigms encompass a different but overlapping subset of elements (Bodien et al., 2024). EEG correlates of semantic processing are known to have prognostic value: examples include the N400 event-related potential (ERP) to incongruent sentence endings (Steppacher et al., 2013), and the rhythmic modulation of EEG responses phase-locked to modulation at word, phrasal and sentential linguistic levels (Gui et al., 2020). Unfortunately, the stimuli used by these studies are artificially manipulated and the meaning of each stimulus is disconnected from the next, which makes listening both jarring and monotonous—and hence, susceptible to inattention.

Recent approaches have begun to focus on natural-language story listening (O'Sullivan et al., 2015). EEG correlates of auditory processing, while listening to Lewis Carroll's *Alice In Wonderland*, were similar between healthy subjects and patients with CMD (Braiman et al., 2018). New signal-processing methods have been developed for extracting responses that are specific to semantic content in natural language—in non-brain-injured controls, these responses are exquisitely sensitive to the subject's understanding of meaning in natural speech (Broderick et al., 2018, 2022).

We apply these novel methods to two adolescents with DoC resulting from severe brain injuries sustained as children. We have previously demonstrated CMD in these subjects based on positive motor command-following responses (Kim et al., 2022b,a); we also showed supporting evidence from ERPs to classical, discrete, auditory language stimuli (Kim et al., 2022a). Here, we derive acoustic and semantic processing markers from natural-language stimuli using methods that estimate temporal response functions (TRFs) (Crosse et al., 2016). The acoustic TRF reflects perceptual and attentional aspects of auditory processing; it contains analogs of the N100 and other event-related potentials that are more familiarly seen in discrete-stimulus designs (Martin et al., 2008). The semantic TRF contains a distinctive component that is spatio-temporally similar to the N400 event-related potential—here, we will include both the N400 and its TRF analog under the umbrella term *meaning-induced neural dynamics* (MIND).

## 2 Methods

### 2.1 Participants

In this paper, we report the data of two adolescent participants with DoC following severe brain injury. Participant P1 had experienced a traumatic brain injury complicated by hypoxemia and associated cardiac arrest at age 9. P1 participated in the current study during a single testing session, at age 16. Participant P2 experienced anoxic brain injury secondary to cardiac arrest at age 13. P2 participated in two separate testing sessions: the discrete semantic paradigm (discussed in detail in Section 2.3.3) was collected at age 18, and the remaining paradigms (discussed in Section 2.3) were collected at age 21. Assessments with the Coma Recovery Scale-Revised (CRS-R) (Giacino et al., 2004) were consistent with vegetative state (P1's total score was 7; P2's total score was 6 in both assessments).

Both participants were previously included in two studies (Kim et al., 2022b,a); they were labeled as participants C and E in Kim et al. (2022b) and as P1 and P2 in Kim et al. (2022a). In the study Kim et al. (2022b), we reported two EEG tests as part of a larger cohort: auditory evoked potentials (AEPs) in simple auditory paradigms, and correlates of motor command-following in oscillatory EEG components (MCF-EEG). In the associated case-series publication (Kim et al., 2022a), we provided a multi-modal profile for P1 and P2, including fMRI motor command-following (MCF-fMRI), EEG correlates of discrete semantic comprehension (N400), fluorodeoxyglucose positron emission tomography, structural MRI, and clinical histories along with the previously-reported ERP and MCF-EEG findings.

All procedures were approved by the institutional review board (IRB) of Weill Cornell Medicine. Parental consents were obtained as per IRB protocols.

### 2.2 Data acquisition

EEG data were recorded with a 128-channel HydroCel Geodesic Sensor Net (EGI, Eugene, OR) (Tucker, 1993). In P1, gel-based sensors were used; in P2, saline sponge and gel-based sensors were used. The impedance of all electrodes was < 75 kΩ at the beginning of the recording as per the manufacturer's specifications. The signals were recorded at 1000 Hz. A 2-piece speaker system was located at 45° (left/right of the midline) at a distance of 57 cm to the ears. Each speech stimulus was normalized to a volume of 70 dB SPL. Stimulus presentation and data acquisition were conducted using the BCI2000 software platform (Schalk et al., 2004).

### 2.3 Stimuli and tasks

We estimated an acoustic and a semantic TRF from the EEG elicited by continuous natural-language listening. We compared these responses against those from the discrete ERP-based paradigms. The paradigms used in this study are described below.

#### 2.3.1 Discrete “clicks” paradigm

For P1, our assessment of auditory processing comes from a click presentation paradigm, in which a rapid sequence of click sounds is designed to elicit an auditory evoked potential (AEP). This paradigm requires no participation beyond passive listening. The click stimuli were a train of 300 biphasic square pulses each with a duration of 1 ms, presented every 745 ms ± 15 S.D.

#### 2.3.2 Discrete “beeps” paradigm

For P2, our assessment of auditory processing was derived from responses to “standard” stimuli in an auditory oddball paradigm (Polich, 2007). This paradigm has frequently been used in both adults and children—including children with brain injury (Kim et al., 2022b; Duszyk et al., 2019)—to assess early attentional processing. During this paradigm, the participant listens to a sequence of abrupt, frequent standard stimuli mixed in random order with rarer deviant stimuli. This task requires no participation beyond passive listening. For the purposes of the current analysis, we consider only the responses to the 270 “standard” stimuli, which comprised about 66% of the stimuli and which were square-wave beeps of 340 ms duration with a fundamental frequency of 400 Hz, repeated with a period of 1 second. Further details about the stimuli used in this paradigm can be found in Kim et al. (2022b).

#### 2.3.3 Discrete N400 paradigm

To estimate P1 and P2's discrete semantic ERPs, we used the sentences from the classic N400 paradigm (Bloom and Fischler, 1980) that are composed of 6–8 words (Connolly and Phillips, 1994). For P1 the stimulus total duration was around 11 min and consisted of 60 congruent sentences (e.g., “Apples and cherries are a type of fruit.”) and 60 incongruent sentences (e.g., “A wild pig is called a shirt.”). For P2, the stimulus total duration was around 6 minutes and consisted of 40 congruent and 40 incongruent sentences. More details can be found in Kim et al. (2022a).

#### 2.3.4 Continuous natural language paradigm

During this paradigm, the participants listened to continuous age-appropriate natural language stimuli, as an audiobook. For P1, we presented 5 min and 40 s of *Yertle the Turtle and Other Stories* by Dr. Seuss read by an adult female reader. For P2, we used around 14 min of *Judy Moody Gets Famous*! by Peter H. Reynolds read by an adult male reader; this was divided into four sections of equal length, with pauses of a few seconds added between sections.

### 2.4 Data processing

Data were processed offline using the MNE package for Python (Gramfort et al., 2013). First, the EEG signals were re-referenced to the average of the right and left mastoids and down-sampled to 250 Hz (after applying an anti-aliasing filter). The signals were then visually inspected by an expert, who marked bad channels (which were then removed and reconstructed via linear interpolation) and bad data segments (which were removed from the analysis in the case of discrete stimuli only). Afterward, the signals were high-pass filtered with a cut-off frequency of 1 Hz. Undesired artifacts from blinks, cardiac activity, and muscle contractions were removed by rejecting the corresponding sources from an independent component analysis (ICA) decomposition using the Infomax algorithm (Bell and Sejnowski, 1995) and projecting back into the sensor space. Lastly, a low-pass filter at 8 Hz was applied to the cleaned EEG signals as in Broderick et al. (2018) and O'Sullivan et al. (2015).

### 2.5 Discrete stimuli analysis

The discrete stimuli (clicks, beeps, and N400 paradigms), elicit an event-related potential (ERP). For this analysis, the data were segmented into epochs time-locked to the onset of the target sound stimuli. For the clicks and beeps paradigms, epochs spanned 100 ms pre-stimulus and 500 ms post-stimulus. For the N400 paradigm, epochs spanned 200 ms pre-stimulus and 800 ms post-stimulus. Each epoch was baseline-corrected by subtracting the mean voltage over the pre-stimulus interval. For the discrete acoustic paradigms (clicks and beeps), the epochs were then processed by the MNE implementation of autoreject (Jas et al., 2016, 2017) which identified and rejected bad epochs (autoreject was not used for the N400 paradigms due to the small number of epochs of this paradigm 60 congruent and 60 incongruent for P1, 40 congruent and 40 incongruent for P2). The ERP was obtained from each session by averaging across epochs at each time point.

### 2.6 Continuous stimuli analysis

Linear de-convolution techniques were used to estimate temporal response functions (TRFs) (Crosse et al., 2016) that reflected the brain's response to continuous stimuli. Separate analyses estimated TRFs from the acoustic and semantic features of the stimuli, with the goal of extracting evidence for both “hearing” and “understanding”. We used a Python implementation of the TRF algorithm developed in our lab and inspired by the one made public by Crosse and colleagues (Crosse et al., 2016; Steinkamp, 2023; Bialas et al., 2023).

#### 2.6.1 Acoustic TRF (A-TRF)

The acoustic TRF was obtained by relating an acoustic representation of the stimulus to the EEG signal. First, the left and right channels of the audio file were averaged to obtain one sound signal. As proposed in the mTRF Toolbox (Crosse et al., 2016, 2021), we extracted the speech envelope directly from the sound signal, after down-sampling to match the EEG sampling rate of 250 Hz, by computing the root-mean-square (RMS) over a window of samples and applying a logarithmic compression factor of 0.3 as in Crosse et al. (2021) and Biesmans et al. (2016). We report the A-TRF using the forward model, which aims to derive a linear de-convolved mapping from the stimulus to each of the EEG channels independently. Thus, one can plot the spatial representation of these models across the scalp—akin to the kinds of topographic analyses that are common in ERP research (see Crosse et al., 2016 for more details). The TRFs are then found by multiple linear regression, with a regularization parameter λ set to 10^5^ (a value that we had been found to be a reasonable one-size-fits-all setting from similar data sets).

#### 2.6.2 Semantic TRF (S-TRF)

The semantic TRF was obtained by relating a semantic representation of the stimulus to the EEG signal. Here, we use the lexical *surprisal* index from each word of the presented stimuli: each word was represented by its conditional probability of occurrence given the word sequence that preceded it, calculated using a large language model—specifically, the 12-layer, 117M parameter version of GPT-2 (Radford et al., 2019). Since brain responses tend to be larger for unexpected words compared to expected words (Kutas and Hillyard, 1984; Mesik et al., 2021), surprisal was represented as an impulse at the onset of each word, where the impulse height was equal to the negative log of the conditional word probability. This leads to less-expected (more informative) words receiving higher surprisal values (Mesik et al., 2021). For this analysis, we employed the forward modeling approach, where the spatial patterns were obtained using equation (5) from Crosse et al. (2016). The regularization parameter in the linear regression was set as λ = 10^5^.

### 2.7 Spatial and temporal range of interest

For the discrete and continuous paradigms, we computed the acoustic and semantic responses from Cz and Pz, respectively, as in published studies (Kim et al., 2022b; Broderick et al., 2018; Gonzalez-Heydrich et al., 2015; Takeshita et al., 2002; Chalehchaleh et al., 2024). For the acoustic paradigms, we noted the latency of the largest negative peak within the range of 80–300 ms (Sussman et al., 2008; Lightfoot, 2016; Stapells et al., 2002). We used a wide range for identifying the auditory evoked potential since a longer-latency response (around 200 ms) is expected in children younger than 12 (Sussman et al., 2008; Lightfoot, 2016; Stapells et al., 2002) and this had previously been found to have persisted into adolescence for P1 (Kim et al., 2022a). For the semantic paradigms, we identified the MIND component as the largest negative peak within 250-500 ms, by analogy with the N400 event-related potential (Kutas and Federmeier, 2000; Kutas and Hillyard, 1980; Camarrone and Van Hulle, 2019).

### 2.8 Statistical analysis

#### 2.8.1 Discrete stimuli

**Figure 1:**
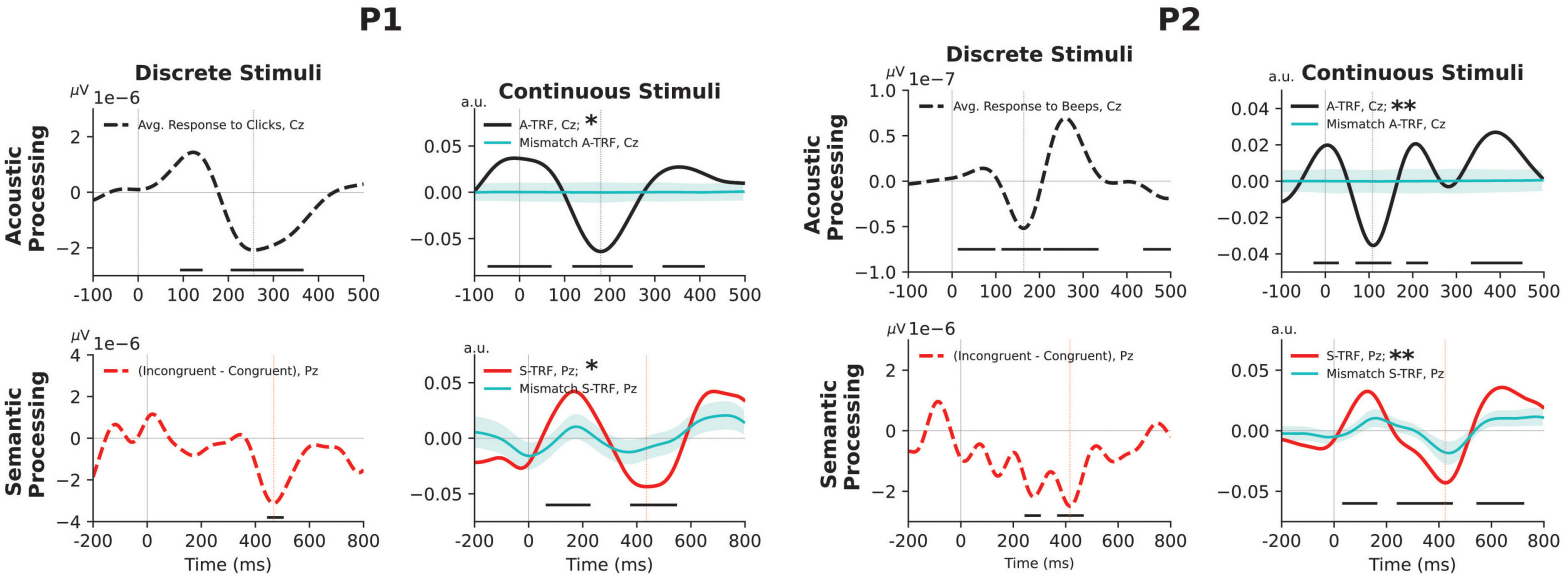
Event-related EEG markers of acoustic and semantic processing of discrete and continuous stimuli for P1 **(left)** and P2 **(right)**. Top-left subplots: average auditory evoked potential recorded from channel Cz (from “click” stimuli in the case of P1, and 340 ms “beep” stimuli for P2). Top-right subplots: acoustic TRF response, estimated at channel Cz. Bottom-left subplots: average difference between responses to incongruent and congruent stimuli in the classical ‘N400' paradigm, at channel Pz. Bottom-right subplots: TRF response correlated with lexical-surprisal feature values at channel Pz. Dotted vertical lines indicate the latency of the largest negative peak (Figure 2 shows the corresponding topographic scalp maps). For discrete stimuli, time points at which event-related potentials were significantly different from zero (uncorrected *p*-value < 0.05) are indicated with a black horizontal line. For continuous stimuli, asterisks in legends denote the overall statistical significance level: *for *p*-values < 0.05 and **for *p*-values < 0.01. Additionally, we show the mean of the re-permuted TRFs (see Section 2.8.2) as cyan traces, and their mean ± one standard deviation as cyan-shaded regions: this is a visualization of the distribution of the TRFs under the null hypothesis that stimulus ordering does not influence the response.

We performed a one-sample two-sided t-test at each time point across trials for both the “clicks” and “beeps” paradigms to identify when the ERP response was significantly different from zero. For the classic “N400” paradigm, we performed a two-sample two-sided t-test at each time point across trials to determine when the incongruent response significantly differed from the congruent response. Statistically significant time points (*p* < 0.05, uncorrected) are shown as black horizontal lines in Figure 1.

#### 2.8.2 Continuous stimuli

Adapting the approach of Chalehchaleh et al. (2024) and Synigal et al. (2023), we established significance in two ways:

(1) To test the presence/absence of a stimulus-specific response, we performed a permutation test on overall prediction accuracy: for both the acoustic and semantic paradigms, we generated 1000 re-permuted versions of the stimuli—either by randomly shifting the starting time (for acoustic features) or by shuffling the numerical values of the lexical surprisal features at the fixed time offsets of each word (for semantic features). For each re-permuted instance, we computed a TRF and calculated its cross-validated prediction accuracy (R)—this was a value ranging from -1 to 1 that estimated how well the computed TRF weights would predict unseen brain responses given corresponding stimulus information. We computed a *p*-value as the proportion of R values from the 1,000 re-permuted instances that exceeded the R of the original stimulus. We use asterisks in the legends of Figure 1 to denote this overall statistical significance level: *for *p*-values < 0.05 and **for *p*-values < 0.01.
(2) To visualize relevant parts of the waveform, we compared against the TRFs computed from re-permuted stimuli at each time-sample of the TRF waveform. Note that the TRF waveforms from the original stimuli, and from the 1,000 re-permuted stimuli, have arbitrary scaling when first computed. Therefore, each waveform was scaled so that the absolute extent of its largest positive or negative peak was equal to the respective overall R. The rescaled re-permuted TRFs were then compared time-point by time-point with the waveform from the rescaled original TRF. We use black horizontal lines in Figure 1 to indicate time periods where the original TRF significantly differed (*p* < 0.05, uncorrected) from the re-permuted TRFs.

## 3 Results

Figure 1 shows the acoustic and semantic responses to discrete stimuli and continuous stimuli for participant P1 and participant P2.

For P1, we observed a negative peak at 256 ms in the acoustic response to discrete stimuli, and at 180 ms for the acoustic TRF in response to continuous stimuli. Though the responses are not identical, they agree substantially as regards temporal response morphology, peak latency, and spatial localization. The acoustic response appears to be more characteristic of children under 12 (Sussman et al., 2008) than of P1's actual age at testing—it is possible that P1's brain injury before age 12 could have influenced the full maturation of the auditory cortex as previously noted (Kim et al., 2022b,a). P1's semantic response exhibits a negative peak at 472 ms for discrete stimuli (obtained using classical N400 paradigm) and 388 ms for continuous stimuli (obtained using semantic TRF).

For P2, we observe a negative peak at 164 ms for the discrete acoustic response and at 108 ms for the continuous acoustic response. We observe semantic responses with a latency of 416 ms for discrete stimuli and 440 ms for continuous stimuli.

**Figure 2:**
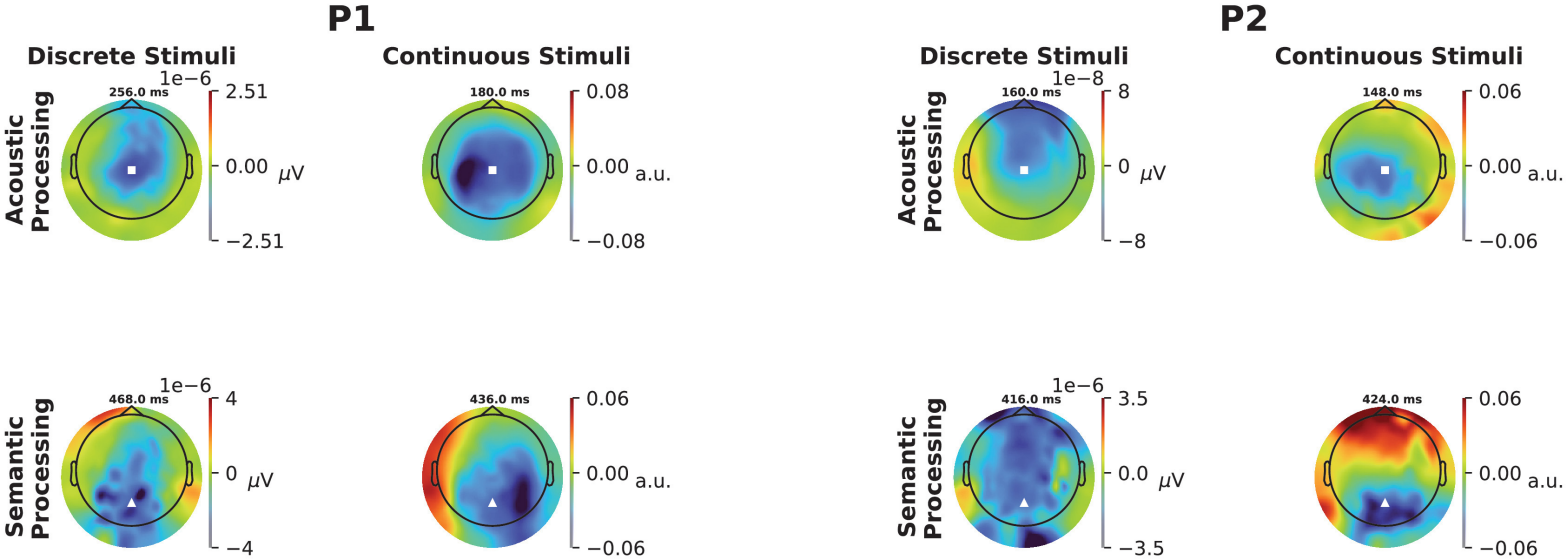
Topographic scalp maps of the ERP and TRF responses to discrete and continuous stimuli for P1 **(left)** and P2 **(right)**. Each map is computed at the time lag corresponding to the largest negative peak marked by a dotted vertical line in the corresponding panel of Figure 1. The locations of Cz and Pz are marked as white squares and triangles, respectively.

For both participants, and both continuous and discrete stimuli, scalp maps (Figure 2) show dominant negative responses over central scalp for acoustic features, and over parietal scalp for semantic features. Permutation tests confirmed the overall significance of both participants' acoustic TRFs by rejecting the null hypothesis that goodness-of-fit is unaffected by the time ordering of the acoustic features, with *p* = 0.0055 and *p* = 0.0005 for the two participants, respectively. Additional permutation tests confirmed overall significance of the semantic TRFs by rejecting the null hypothesis that goodness-of-fit is unaffected by the ordering of the contextual-meaning-derived information values of the words, with *p* = 0.0175 and *p* = 0.0035 for the two participants, respectively. (Further details about the permutation tests are provided in Section 2.8.2, above; see also the caption of Figure 1).

## 4 Discussion

We have highlighted a new method for deriving correlates of acoustic processing and semantic comprehension that agree with, but go beyond, those of classical methods. In two adolescents in a disorder of consciousness following severe brain injury, we have shown evidence of preserved processing of auditory and semantic information. The acoustic and semantic results from the continuous stimuli are consistent with previous studies; displaying prominent negativity at around 100–300 ms over central scalp (Crosse et al., 2016; Sussman et al., 2008) for the former, and 400 ms over parietal scalp (Broderick et al., 2018; Kutas and Federmeier, 2000) for the latter. Consistent with what has been reported before in healthy adults (Broderick et al., 2018), there is a strong correspondence between responses from discrete and continuous stimuli.

We previously reported that the two participants in this study met diagnostic criteria for cognitive motor dissociation (CMD). This was based on their positive EEG responses to the commonly-used task of attempted motor command-following, indicating covert volitional engagement. Our current findings—evidence of covert semantic processing—enrich their CMD diagnosis by providing further supporting evidence of covert cognition from a complementary cognitive task.

The meaning-induced neural dynamics (MIND) identified in the semantic TRF (S-TRF), and our statistical tests of their overall significance, are the basis for our strongest claims of preserved cognition. Not only do these participants' N400 waveforms and analogous MIND peaks in the S-TRF qualitatively resemble those of healthy subjects (Broderick et al., 2018, 2022), they also depend specifically on the semantically-coherent ordering of the words. This is because the S-TRF is estimated by regression against *surprisal*, a measure of each word's informational contribution given its preceding context. Broderick et al. (2022) demonstrated experimentally that the S-TRF MIND depended on the stimulus text having coherent meaning, rather than being a non-specific word-level response: the peak deteriorated as stimulus word order was increasingly scrambled. In the current study, our permutation tests perform a similar scrambling, but on the analysis side: they simulate the null hypothesis that the same set of surprisal values, time-locked to the same set of word onset times, would produce equally well-fitting and predictive S-TRF results regardless of their ordering. We were able to reject this null hypothesis for both participants, indicating that the specific ordering of word meanings in the stimulus text was crucial for recovering the observed S-TRF from their EEG. For these participants' families, this is the strongest evidence to date that the participants' brains were capable of processing spoken word meanings in context.

Using the brain's response to continuous natural language stimuli as a proxy measurement of cognitive function offers many benefits. First, it offers a feasible method for deriving correlates of sensory and semantic processing in patients remaining in a DoC, requiring low effort from the patient—*i.e*., only the effort of listening to a story in contrast to the sustained attention and arousal control that is required in mental-imagery tasks, or in listening to monotonous, meaningless stimuli. This new method is no less feasible compared to traditional methods in that it does not require longer sessions. In fact, a semantic response can be estimated from a single minute of continuous data alone (O'Sullivan et al., 2015; Broderick et al., 2018), offering the potential for more-granular analyses that track variations in attention and arousal through the session. Second, because of the limitless corpus of potential stimulus material, multiple repeats of this paradigm can be employed without repeating the stimulus; in people recovering from brain injury, tracking residual and emerging functions can therefore be conducted while keeping the test engaging. Third, this method has been used in adult non-brain-injured controls to detect attention in the presence of competing stimuli (O'Sullivan et al., 2015); when employed in this manner, it has the potential to assess higher-order cognitive abilities, *e.g*. executive attention. Fourth, this method can be employed in very young children for whom methods requiring more cognitive resources such as motor command-following may be infeasible (Souto et al., 2020). Fifth, the complexity and genre of the material can be tailored according to the patient's age and interests; this not only allows accommodation of a wide range of situations but has the potential to allow complexity to be varied to gauge the degree of language recovery. Lastly and most importantly, assessing the brain's response while hearing a story has the potential of identifying cognitive recovery across a continuum beginning with sensory awareness of sounds through higher-order processing of semantics. This is expected to identify patients who have recovered some, but not all, cognitive abilities. This may include, but may not necessarily be limited to, patients identified as having metabolic preservation in the fronto-parietal network, who are referred to as MCS* (Thibaut et al., 2021).

In conclusion, while studies have reported on discordance between bedside function and covert-command-following, there is no knowledge of the range of cognitive function that may be hidden by motor impairment. Our new tool offers a way to assess a non-verbal person's speech processing abilities hierarchically (from lower-level sensory responses to processing of higher-level semantic features), and to grade their comprehension (by comparing responses to material of varying complexity). As such, we expect this method can be applied more generally to identify and characterize covert cognition both within and beyond the context of cognitive-motor dissociation.

## 5 Limitations

While some normative EEG data from adults are available and relevant to our participants, who were in late adolescence to early adulthood, the lack of age-matched healthy pediatric controls limits our ability to fully contextualize the observed neural responses. The discrete N400 does not differ drastically between adolescents and adults (Cummings et al., 2008)—though we might expect the same to be true of the analogous MIND in the semantic TRF, this remains to be confirmed. Future research should aim to develop normative TRF datasets in healthy children and adolescents to strengthen interpretability in clinical populations.

## Data Availability

Data and stimuli from this study are available from the corresponding author upon reasonable request, subject to data sharing agreements that comply with institutional requirements and the consent of participating families.
